# Evaluating of HPV–DNA ISH as an adjunct to p16 testing in oropharyngeal cancer

**DOI:** 10.2144/fsoa-2020-0052

**Published:** 2020-08-03

**Authors:** Jeffrey Chi, Isabel R Preeshagul, Silvat Sheikh-Fayyaz, Sewit Teckie, Nina Kohn, Yonah Ziemba, Alice Laser, Douglas Frank, Maged Ghaly, Dev Kamdar, Dennis Kraus, Doru Paul, Nagashree Seetharamu

**Affiliations:** 1 Department of Medical Oncology/Hematology, Donald & Barbara Zucker School of Medicine at Hofstra/Northwell, New York, NY 11042, USA; 2 Memorial Sloan Kettering Cancer Center, New York, NY 10065, USA; 3 Department of Pathology, Donald & Barbara Zucker School of Medicine at Hofstra/Northwell, New York, NY 11042, USA; 4 Department of Radiation Medicine, Donald & Barbara Zucker School of Medicine at Hofstra/Northwell, New York, NY 11042, USA; 5 Department of Biostatistics, Donald & Barbara Zucker School of Medicine at Hofstra/Northwell, New York, NY 11030, USA; 6 Department of Otolaryngology – Head & Neck Surgery, Donald & Barbara Zucker School of Medicine at Hofstra/Northwell, New York, NY 11042, USA

**Keywords:** agreement level, concordance, HPV detection, *in situ* hybridization, oropharyngeal squamous cell carcinoma, p16 immunochemistry

## Abstract

**Aim:**

Current guidelines recommend p16 immunohistochemistry (IHC) for testing human papillomavirus (HPV) in oropharyngeal carcinoma (OPSCC). We evaluated the value of adding DNA *in situ* hybridization (ISH) to p16 IHC.

**Methods:**

Fifty patients with OPSCC were analyzed. Concordance between HPV–DNA ISH and p16 IHC was measured by Gwet's agreement coefficient.

**Results:**

p16 IHC was positive in 35/48 (72.9%), negative in 8/48 (16.7%) patients. Wide spectrum DNA–ISH was positive in 9/23 (39%) and negative in 14/23 (60.9%) patients. High-risk 16/18 (HR) HPV DNA–ISH was positive in 11/23 (47.8%) and negative in 12 (52.2%) patients. The agreement between HPV DNA–ISH and p16 IHC is fair (Gwet's AC1 = 0.318).

**Conclusion:**

The agreement between p16 IHC and HPV–DNA ISH was fair. However, ISH sensitivity was low. Our findings add to the current data that p16 IHC testing is reliable and may be enough as a stand-alone test for HPV detection in OPSCC.

## Lay abstract

Current testing guidelines recommend p16 immunochemistry (IHC) for detecting human papillomavirus (HPV) in oropharyngeal cancer (OPSCC). We evaluated the value of adding HPV DNA *in situ* hybridization (ISH) to p16 IHC. Fifty patients with OPSCC were analyzed. p16 IHC was positive in 72.9% of patients and DNA–ISH was positive in 39% of patients when wide spectrum probe was used and positive in 47.8% of patients when high-risk probe was used. The agreement between the two tests was fair. However, DNA–ISH sensitivity was low. p16 IHC may be enough as a stand-alone test for detection of HPV in OPSCC.

Head and neck cancers are the sixth most common malignancy worldwide with a global incidence of over 650,000 cases annually and over 330,000 deaths [1]. Over 92,000 of these cases are oropharyngeal squamous cell carcinomas (OPSCC). While head and neck cancer associated with smoking and alcohol use are declining, the incidence of cancers caused by human papillomavirus (HPV) is increasing. Based on cohort studies from western hemisphere, approximately 50% of cases from 1990s were attributable to HPV whereas 70% of OPSCC cases today are thought to be associated with HPV, most frequently types 16 and 18 [2]. In the United States, the estimated incidence of HPV-associated OPSCC was 4.62 per 100,000 versus 1.81 for HPV-negative OPSCC [3].

HPV-associated OPSCC is biologically and clinically distinct from tobacco and alcohol-related OPSCC. Histologically, HPV-associated OPSCC are characteristically nonkeratinizing and basaloid whereas HPV-negative OPSCC is classically keratinizing [4,5]. HPV-associated OPSCC are usually diagnosed in advanced stages due to small primary tumor size and early nodal metastasis. However, the prognosis and clinical outcomes of these patients are excellent with 80% or higher three-year overall survival for locally advanced disease [2,6]. The oncogenic potential of high-risk (HR) HPV is related to transcription of oncoproteins E6 and E7. E6 promotes the ubiquitination and proteasomal degradation of p53 preventing induction of apoptosis when cells enter unscheduled S phase. E7 oncoprotein promotes degradation and inactivation of the tumor suppressor retinoblastoma protein (pRb) and promotes the transcription of p16. In normal cells p16 acts as tumor suppressor by inhibiting CDK4/6 which lead to accumulation of pRb protein and cell cycle arrest. However, this process is subverted in HPV-associated tumor by E7 targeting and proteosomal degradation of pRb. Inactivation of pRb and overexpression of p16 play an important role in the survival of HPV driven tumor cells (Figure 1) [7,8]. This also highlights the applicability of p16 IHC as a surrogate marker for HPV positivity.

**Figure 1.**
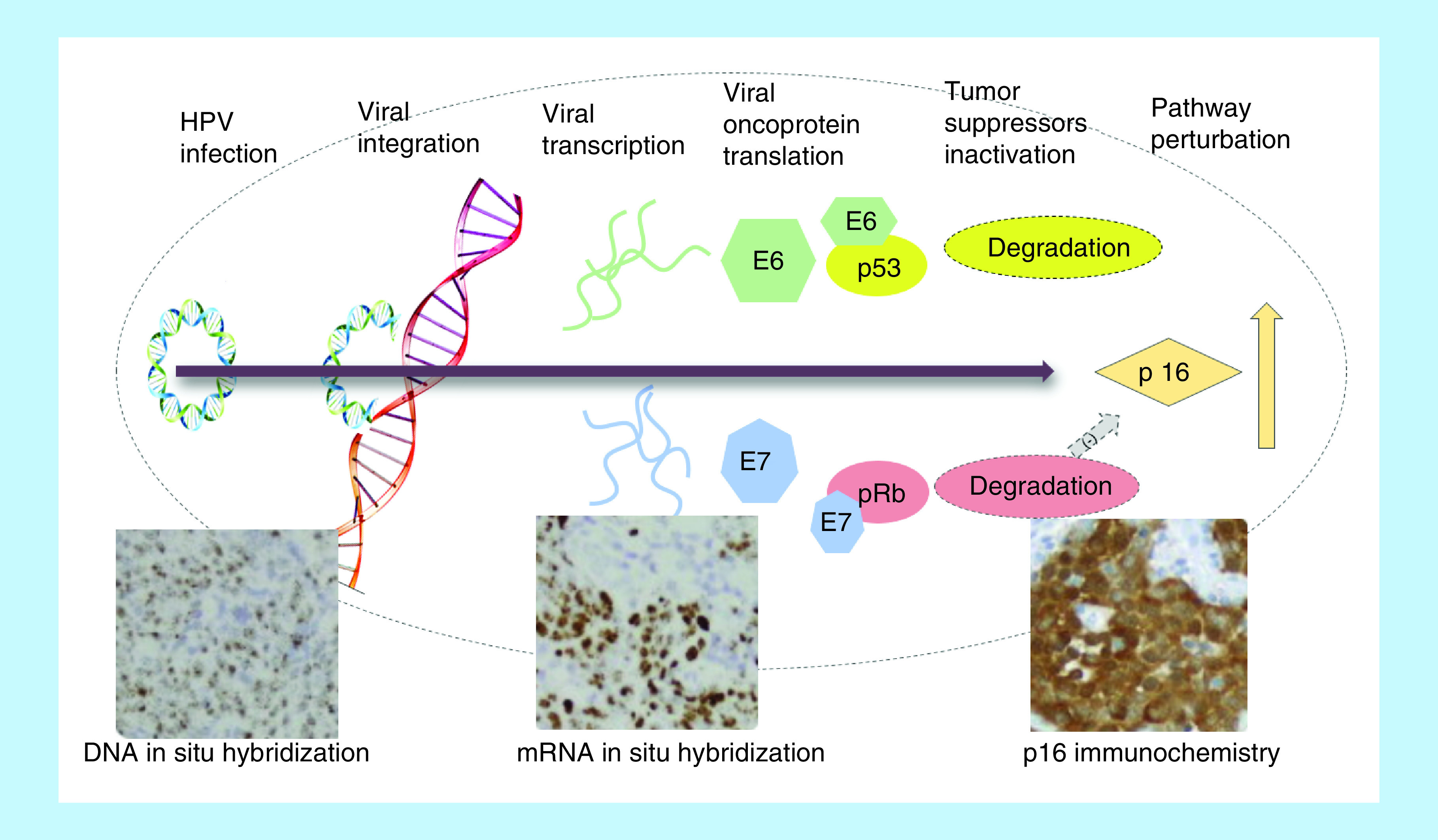
HPV infection and the process of malignant transformation. HPV: Human papillomavirus.

Because of the clear prognostic importance HPV status in patients with OPSCC, the eighth edition of the AJCC Cancer Staging Manual has separated HPV positive OPSCC from HPV negative OPSCC. Clinical trials are looking at de-escalation strategies (e.g., NCT01855451, NCT01932697) for HPV positive disease and it is possible that treatment algorithms in the near future may heavily depend on HPV status. Therefore, it is of paramount importance to accurately categorize OPSCC as HPV positive or negative. However the optimal method(s) for testing HPV status is still not completely standardized. The current guidelines from the College of American Pathologists (CAP) as well as American Society of Clinical Oncology (ASCO) recommend testing for HPV in tumor samples of OPSCC using surrogate marker p16 IHC [9,10]. Performing subsequent confirmatory HPV-specific tests is at the discretion of the pathologist. Previously, studies have variably used HPV-specific tests such as HPV DNA–ISH, DNA polymerase chain reaction (PCR), mRNA RT PCR and mRNA ISH for viral oncoproteins E6 and E7, as well as IHC staining of HPV surrogate marker p16 (Figure 1). The detection of HPV E6/E7 mRNA by PCR is considered the gold standard. However, the test cannot be performed directly on the readily available formalin-fixed paraffin-embedded (FFPE) specimens and requires cumbersome preparations of the tissue sample, making it impractical for use in most clinical context. A more recently available test, HPV E6/E7 mRNA ISH which can be performed on FFPE specimens has been validated against PCR, HPV–DNA ISH and p16 IHC and shown to have high concordance rate with p16 IHC in OPSCC [11]. This test, however, is also not widely available and hence, cannot be used for large multinational studies or routine clinical practice. For these reasons, p16 IHC and HPV–DNA ISH, which can be performed on readily available FFPE tissues samples are the widely used testing modalities for HPV detection.

In our study, we evaluated the utilization and value of using HPV–DNA ISH as an adjunct to p16 IHC to detect HPV status of oropharyngeal carcinoma in our practice. We also assessed the level of concordance between two types of tests with a special focus on never-smokers, in whom majority of the tumors can be considered HPV positive [12] (Figure 2).

**Figure 2.**
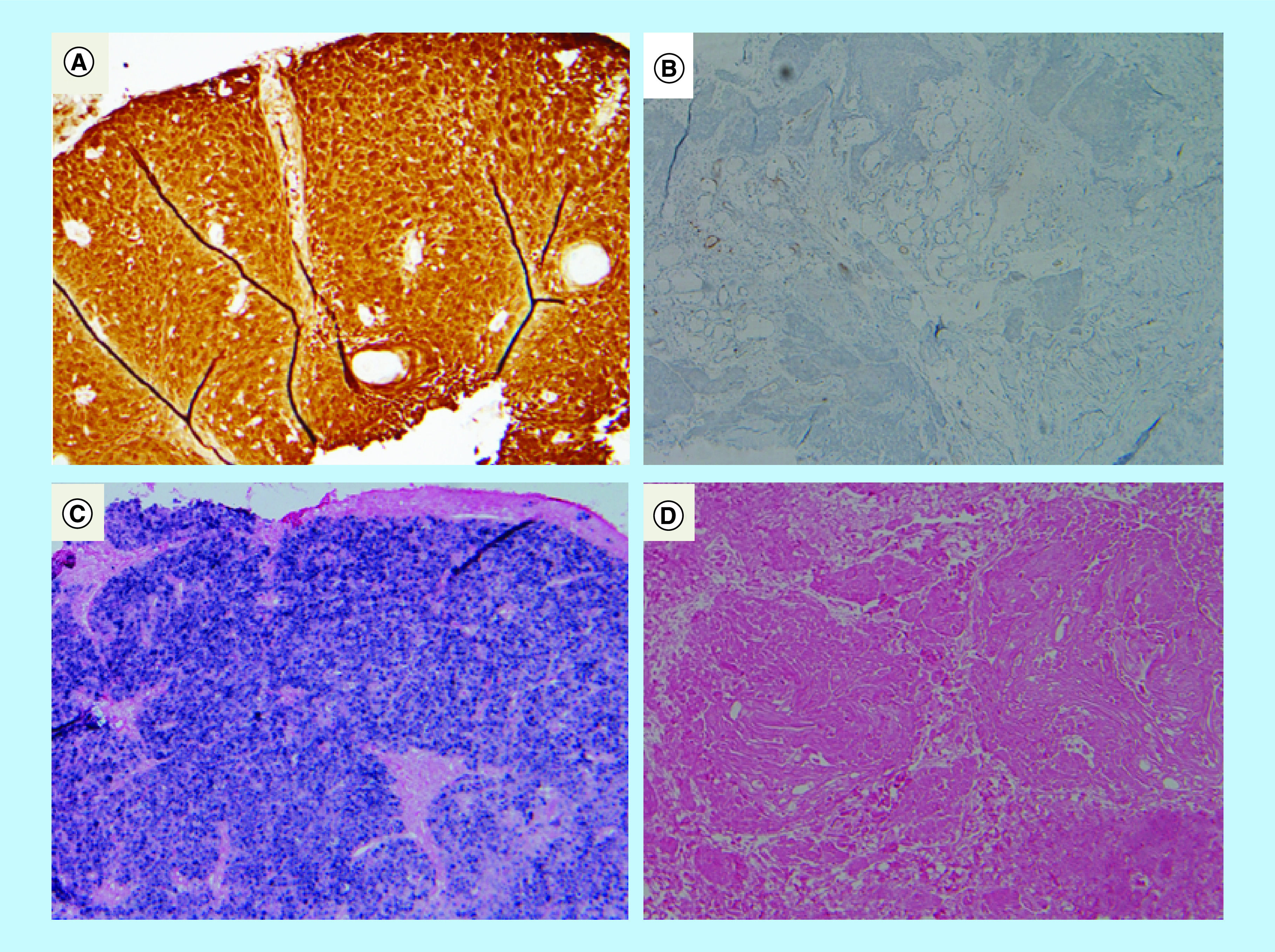
p16 Immunohistochemistry and HPV DNA *in situ* hybridization of formalin-fixed paraffin-embedded specimens.

## Materials & methods

A retrospective analysis of 50 patients with OPSCC diagnosed between 2012 and 2014 was conducted. The study was approved by Institutional Review Board of North Shore University Hospital. Study subjects were identified through our hospital cancer registry. Data points included age, gender, smoking history, stage of disease at diagnosis, primary site of disease and HPV status. All p16 IHC tests were performed at our own institution and were reviewed by board certified pathologists. Immunohistochemical staining for p16 was performed using Ventana BenchMark Autostainer (Ventana Medical System, AZ, USA). Selected paraffin blocks from each case were cut at 5-μm thickness and dried at 60°C for 1 hour. Following deparaffinization, rehydration and blockage of endogenous peroxidase, slides were treated with antigen retrieval solution followed by incubation with primary antibodies against p16 (1:100; clone EPR1473, Abcam, MA, USA). Antibody binding was visualized using the iVIEW DAB Detection Kit (760-091). Only samples with strong, diffuse nuclear and cytoplasmic staining greater than 70% were regarded as positive. HPV-specific tests, HR DNA–ISH or wide-spectrum (WS) DNA ISH probes that target the genomic DNA of HPV types 6, 11, 16, 18, 30, 31, 33, 35, 45, 51 and 52, were performed at GenPath Diagnostics and Integrated Oncology. The probe used was Leica-Kreatech HPV ISH probes on Leica III BOND autostainer. The HPV positivity was defined as punctate and diffuse nuclear staining of tumor cells. There were variabilities in the selection of HPV–DNA ISH tests, however all of tests included probes against HR HPV ISH types 16/18. The variability in the selection of HPV ISH probe is because of unclear standardized testing guidelines, multiple tests available on the test directory made it difficult to maintain consistency among different ordering professionals and referring sites with less experience ordering the tests.

## Statistical analysis

Descriptive statistics were used to summarize the data (median and range for age, and frequency and percent for categorical data). The level of agreement between p16 IHC and HPV–DNA ISH (WS and HR) was measured by calculating Gwet's agreement coefficient (AC1). All analyses were carried out using SAS Version 9.4 (SAS Institute Iinc. NC, USA)

## Results

Fifty patients were analyzed. Thirty-nine were male (78%) and median age was 61.5 years. Most common primary site was base of tongue (n = 28; 56%) followed by tonsil (n = 19; 38%). Most patients had advanced disease: 16 (32%) stage III, 31 (62%) stage IVA, 1 (2%) stage IVB. Patient demographics are shown in Table 1.

**Table 1.** Demographic, clinical and pathologic characteristics by group.

Group (N)	All (50)
Age, median (range)	61.5 (41–81)
Sex (%):	
– Male	39 (78)
– Female	11 (22)
Smoking (%):	
– Yes (current or former)	30 (60)
– No (never)	20 (40)
Alcohol drinkers (%):	
– Heavy drinkers	6 (12)
– Social drinkers	31 (62)
– Never drinkers	13 (26)
Site of disease (%):	
– Base of tongue	28 (56)
– Soft palate	3 (6)
– Tonsil	19 (38)
T Stage (%):	
– I	1 (2)
– II	1 (2)
– III	16 (32)
– IVA	31 (62)
– IVB	1 (2)

p16 IHC testing was performed in 48 (96%) and ISH in 43 (86%). Wide-spectrum (WS) ISH was utilized in 23 (46%) and HR HPV ISH in 23 (46%; three patients had both WS and HR HPV testing). There were two patients who did not have p16 IHC or HPV–DNA ISH testing performed. Thirty five out of 48 patients (72.9%) tested positive for p16 IHC, eight (16.7%) tested negative and five (10.4%) were equivocal. For WS HPV–DNA ISH, 9/23 (39%) tested positive and 14/23 (60.9%) tested negative. For HR HPV–DNA ISH, 11/23 (47.8%) patients tested positive and 12/23 (52.2%) tested negative. p16 IHC was positive in 18/20 (90%) of positive HPV–DNA ISH (WS and HR) tumors and 2/20 (10%) were equivocal whereas out of positive p16 IHC tumor samples, 18/35 (51.4%) were positive for HPV–DNA ISH (WS and HR). The results of p16 IHC and HPV ISH are shown in Table 2.

**Table 2.** p16 immunohistochemistry versus human papillomavirus *in situ* hybridization.

p16 IHC (n)	WS HPV–DNA ISH (n)		16/18 HPV–DNA ISH (n)		ISH not done	Total
	Positive (9)	Negative (14)	Positive (11)	Negative (12)		
Positive (35)	9	8	9	8	1	35 (72.9%)
Negative (8)	0	5	0	3	0	8 (16.7%)
Equivocal (5)	0	1	2	1	1	5 (10.4%)
Not done (2)	0	0	0	0	2	2
Total (50)	9 (39.1%)	14 (60.9%)	11 (47.8%)	12 (52.2%)	4	50 (100%)

HPV: Human papillomavirus; IHC: Immunohistochemistry; ISH: *In situ* hybridization.

Among never-smokers (n = 20), p16 IHC was diffusely positive in 19/19 (100%) tested, whereas HPV ISH was positive in 8/17 (47%) as shown in Table 3.

**Table 3.** Human papillomavirus testing in never-smokers.

Test (n)	Positive	Negative	Equivocal
p16 IHC (19)	19 (100%)	0 (0%)	0 (0%)
HPV ISH (17)	8 (47.1%)	9 (52.9%)	0 (0%)

IHC: Immunohistochemistry; ISH: *In situ* hybridization.

The level of agreement between p16 IHC and HPV–DNA ISH (WS and HR) was assessed using Gwet's AC1 which divides the strength of agreement into poor, fair, moderate, good and very good. In our study, the level of agreement based on Gwet's AC1 was found to be 0.318 (95% CI: 0.017, 0.620) which is fair (AC1 0.2–0.4).

## Discussion

It has now been established beyond any doubt that HPV positivity connotes excellent response to treatment and overall prognosis when compared with HPV-negative OPSCC. Several trials are underway to determine if de-escalation strategies would be appropriate for these patients with goals to reduce toxicity/morbidity from chemotherapy and radiation in these patients while maintaining similar survival rate. With evolving treatment strategies based on results of HPV status of oropharyngeal cancer, standardization and optimization of testing methods are of paramount importance. Indeed, a majority of currently enrolling clinical trials (e.g., NCT01855451, NCT01932697 etc.) utilize p16 IHC as a stand-alone test as part of eligibility criteria. However, there are some that require HPV specific testing to be positive for inclusion (NCT02280811, NCT02163057). Moreover, the current eighth edition of AJCC Cancer Staging Manual classifies OPSCC into p16-associated (HPV associated) and p16-negative disease but does not specify the tests that should be utilized for assessing HPV status.

As our study demonstrates, p16 IHC testing was used fairly consistently (96%) at our institution and our referring sites for assessing HPV status of oropharyngeal cancer while the type of HPV DNA ISH testing varied considerably. Overall, the level of agreement between p16 IHC and HPV–DNA ISH tests was fair but p16 IHC positivity was substantially higher than that of DNA–ISH.

p16 overexpression is widely accepted as a very sensitive surrogate marker for transcriptionally active HPV. However, the main critique for using p16 IHC as a stand-alone test is that p16 is a surrogate marker and the IHC does not directly measure the presence of transcriptionally active HPV. When using p16 IHC as a stand-alone test for risk stratification, there is concern for patients with positive p16 but truly HPV negative tumor with aggressive disease course can be wrongly stratified into lower risk category. However, several studies have shown that there is strong correlation of p16 expression and improved outcomes in multivariate analyses after controlling for variables. Recent observations suggest that expression of p16 in OPSCC portends a favorable outcome regardless of HPV status. Hazard ratios for overall survival in patients with p16 positive OPSCC in recently published studies ranges from 0.21 to 0.42 when compared with p16 negative cases [13–15]. Additionally, there are also studies which show that adding HPV–DNA ISH to p16 does not improve the survival correlation [13,16,17].

In our study, p16 IHC was positive in 100% of the non-smokers, a cohort of patients who are highly likely to be HPV positive [12]. HPV–DNA ISH was positive in only 47% of this group of patients suggestive of its low sensitivity. We acknowledge that our study is subject to several limitations. Data are from a single referral center; it is retrospective in nature; sample size is small and lacks statistical power to make any definitive statements. However, our findings add to the currently available data that p16 IHC is more sensitive test (91%–97%) than HPV–DNA ISH (76–92%) [18] and that p16 IHC may be sufficient as a stand-alone test for determining HPV status in patients with OPSCC.

## Conclusion

As we move closer toward new HPV-specific treatment paradigms in OPSCC, accurate assessment for HPV status is essential. The discordance among currently available HPV testing methods makes it necessary to incorporate not only the HPV status of OPSCC patients but also to specify optimal testing guidelines into the decision-making algorithm. Our findings add to the currently available data that p16 is an easily available, highly sensitive and reliable surrogate marker for HPV positivity. The utilization of p16 IHC as a stand-alone test is reasonable for HPV detection in OPSCC until other testing modalities such as HPV E6/E7 mRNA ISH or detection of E6/E7 mRNA by RT-PCR become widely available. While treatment strategies for oropharyngeal cancer are evolving, it is also imperative that we accurately test and document HPV status since this will hugely impact treatment decision-making.

## Future perspective

Due to the markedly improved outcome of HPV positive OPSCC, many studies with de-intensification treatment strategies are under investigation. The results of these de-intensification trials are highly anticipated and the general hypothesis is that milder treatment strategies offer similar benefit as standard regimens for patients with HPV positive OPSCC while producing fewer treatment-related side effects. It is therefore extremely important to optimize and standardize HPV testing methodologies that are easy to perform, broadly available and cost effective. Addition of HPV specific test, mRNA–ISH as it becomes widely available, to p16 IHC may help optimize testing for HPV status by maximizing sensitivity and specificity. Additional validation studies and cost analyses are needed to standardize testing guidelines for detecting HPV in OPSCC.

**Executive summary**

- The incidence of human papillomavirus (HPV) associated oropharyngeal cancer (OPSCC) is increasing in the Western countries while the incidence HPV-negative (alcohol, smoking related) OPSCC is declining. Today, 70% of OPSCC cases are HPV-associated.
- Compared with HPV-negative OPSCC, HPV-associated OPSCC confers better prognosis despite often diagnosed in advanced stages due to small primary tumor and early metastasis.
- Expression of HPV viral protein E6 and E7 leads to oncogenesis. E6 promotes the degradation of tumor suppressor p53 preventing apoptosis when cells enter unscheduled S-phase. E7 promotes the degradation of tumor suppressor *pRb* and increases the transcription of p16 which leads to tumor cell survival.
- College of American Pathologists (CAP) and American Society of Clinical Oncology (ASCO) recommend testing for HPV in tumor samples of OPSCC using surrogate marker p16 immunohistochemistry (IHC). Performing subsequent confirmatory HPV-specific tests is at the discretion of the pathologist.
- DNA–*in situ* hybridization (ISH) and p16 IHC are widely used testing methods due to their availability and ease of use as they can be performed on the standard formalin-fixed, paraffin-embedded (FFPE) specimens. mRNA–ISH can also be performed on FFPE samples and has high concordance rate with p16 IHC but not yet widely available. The gold standard test is detection of HPV E6/E7 mRNA by RT PCR. However, the test requires cumbersome sample preparation, cannot be performed on FFPE tissue samples and not widely available.
- Accurately diagnosing HPV positivity in OPSCC is important as it confers better prognosis. Numerous de-intensification treatment strategies are under investigation for HPV-associated OPSCC that can potentially reduce treatment related toxicities and morbidities while maintaining similar progression-free survival and overall survival.
- Our study showed that p16 IHC testing was used consistently in 96% of patients whereas the use of HPV DNA–ISH varied (46% wide spectrum probes and 46% high risk probes).
- p16 IHC was positive in 90% of positive HPV–DNA ISH (WS and HR) tumors whereas out the positive p16 IHC tumor samples, 51.4% were positive for HPV–DNA ISH (WS and HR).
- Amongst never-smokers with OPSCC, who have high likelihood of having HPV-associated disease, p16 IHC was diffusely positive in 100% of patients, whereas HPV ISH was positive in 47%.
- The level of agreement between p16 IHC and HPV–DNA ISH (WS and HR) was fair (Gwet's AC1 = 0.318; 95% CI: 0.017, 0620).
- Our study adds to the currently available data that p16 IHC is highly sensitive and reliable surrogate marker of HPV positivity.
- The utilization of p16 IHC as a stand-alone test is reasonable until more sensitive HPV-specific test such as E6/E7 mRNA–ISH becomes more widely available.
